# Protein interaction evolution from promiscuity to specificity with reduced flexibility in an increasingly complex network

**DOI:** 10.1038/srep44948

**Published:** 2017-03-24

**Authors:** T. Alhindi, Z. Zhang, P. Ruelens, H. Coenen, H. Degroote, N. Iraci, K. Geuten

**Affiliations:** 1 Department of Biology, KU Leuven, Leuven, Belgium; 2 Department of Pharmacy, University of Salerno, Salerno, Italy

## Abstract

A key question regarding protein evolution is how proteins adapt to the dynamic environment in which they function and how in turn their evolution shapes the protein interaction network. We used extant and resurrected ancestral plant MADS-domain transcription factors to understand how SEPALLATA3, a protein with hub and glue properties, evolved and takes part in network organization. Although the density of dimeric interactions was saturated in the network, many new interactions became mediated by SEPALLATA3 after a whole genome triplication event. By swapping SEPALLATA3 and its ancestors between dimeric networks of different ages, we found that the protein lost the capacity of promiscuous interaction and acquired specificity in evolution. This was accompanied with constraints on conformations through proline residue accumulation, which made the protein less flexible. SHORT VEGETATIVE PHASE on the other hand (non-hub) was able to gain protein-protein interactions due to a C-terminal domain insertion, allowing for a larger interaction interface. These findings illustrate that protein interaction evolution occurs at the level of conformational dynamics, when the binding mechanism concerns an induced fit or conformational selection. Proteins can evolve towards increased specificity with reduced flexibility when the complexity of the protein interaction network requires specificity.

Whether it is a receptor on the surface of the cell sensing the environment^1^,^2^,^3^,^4^, the movement of muscles^5^, cell differentiation and development^6^,^7^,^8^,^9^, metabolic pathways or switching genes on and off almost every biological aspect of living organisms involves protein-protein interactions^10^,^11^,^12^. The complexity of protein-protein interactions is described using networks and a complete understanding of these networks can only be achieved through knowledge of the chemical and physical properties of the involved proteins. These physicochemical properties determine how proteins fold into the right structure, which parts are involved in the interaction with other proteins and what conditions facilitate or disturb the protein interactions they are involved in. Hubs for instance, proteins that have many interactions, achieve these interactions through a variety of means. Multi-hubs have multiple interaction interfaces, while promiscuous hubs maintain a degree of structural disorder, a different folding upon binding or a nonspecific interaction interface. Date-hubs by contrast interact at different times or locations^13^,^14^.

A valuable tool to further our understanding of protein interactions is to study how they originate and disappear in evolution. This viewpoint allows to answer questions of a different nature. How come some proteins evolve towards molecular promiscuity yet others evolved to be specific^15^,^16^. When does protein flexibility increase in evolution to ensure protein interaction^17^,^18^? In complex protein networks, is there selection on conserving a specific tertiary structure or does selection on protein dynamics exist affecting the energy landscape of complex formation? To help answer these questions we focused on MADS-domain transcription factors, one of the essential protein families in the development of eukaryotic organisms that has been extensively studied in plants^19^,^20^,^21^. (MADS-domain proteins were named after the first members discovered in different species, MCM1 in *S.cerevisiae*, AGAMOUS in *A.thaliana*, DEFICIENS *A.majus* and SRF in *H.sapiens*).

MADS-domain transcription factors are involved in numerous developmental processes in eukaryotic organisms. In plants the MIKC clade plays a key role in regulating reproductive development, including transducing environmental temperature and light signals to regulate flowering time, establishing floral organ identity and regulating seed and fruit development^22^,^23^,^24^,^25^. The MIKC clade contains four distinct domains, a MADS-DNA binding domain, an Intervening domain, a Keratin-like domain and a C-terminal domain, hence MIKC, (see Fig. 1A), Comparative analysis of their sequences in the context of genomes indicates that this gene family amplified through a number of large scale duplication events^8^,^26^,^27^,^28^. After duplication, the duplicated genes are retained by the dosage balance theory, were genes are conserved in equal dosage not to disturb balance in protein network^28^,^29^,^30^,^31^, one scenario is that a copy of the gene becomes redundant, placing it under less negative selection so that it can accumulate neutral mutations to gain a new function or loose an old one. Alternatively, the two copies can partition functions. Such scenarios can explain the retention of the duplicated genes after each major duplication event followed by rewiring of the resulting protein networks^29^,^30^,^31^,^32^,^33^,^34^,^35^,^36^,^37^.

MADS-domain proteins form both homo- and heterodimers and in the floral quartet model^22^,^23^,^38^ they are considered to function as tetramers that recruit DNA regulatory elements to control gene expression through DNA looping^21^,^38^,^39^,^40^,^41^. Some MADS-domain proteins have come to function as hubs, interacting with many other MADS-domain proteins. Other proteins can be considered as islands, having very few protein-protein interactions^26^,^37^,^42^,^43^. Our understanding of the molecular mechanisms involved in positioning these proteins into their protein-protein interaction networks is far from complete. The E lineage protein SEPALLATA3 (SEP3) has been previously found to have both hub properties and glue properties, in that it interacts with many other MADS-domain proteins in dimeric complexes as determined by using yeast-two-hybrid, but also mediates interactions between many protein combinations as a glue, as determined by using yeast-three-hybrid assays^42^,^43^. MIKC-type MADS-domain proteins have a characteristic four domain structure, in which the M-domain functions in DNA binding, the I-domain has no clearly defined one function but it plays a role in dimerization, the K-domain functions in protein-protein interactions through dimer and tetramer formation, and the C-domain again has no clearly defined single function but it can contain a transactivation domain^21^ (Fig. 1A). Recently, a partial crystal structure of the SEP3 keratin-like domain (K-domain) has been published^44^, the crystal structure showed the partial protein in tetramer conformation (Fig. 1B), where the three K-subdomains (K1, K2, K3) form two major α-helices interrupted by a loop allowing them to fold into dimer/tetramer conformations. Two different full-length models of SEP3 dimers can be predicted by combining the K-domain crystal structure with homology modeling of the MADS-domain (M-domain) based on crystal structure templates of either SERUM RESPONSE FACTOR (SRF) or MYOCYTE ENHANCER FACTOR-2 (MEF2)^45^,^46^ (Fig. 1A, right side).

In this study we sought to integrate data from the network level down to the structural level to understand how molecular evolution can explain the evolution of specificity of proteins in their protein interaction networks.

## Results

### The SEP3-mediated network increased in density after the gamma whole genome triplication

In two previous studies, we inferred and resurrected ancestral sequences for plant MADS-domain proteins which made it possible to characterize these ancient proteins using current laboratory methods^37^,^47^. We were able to reconstruct the networks of their protein interactions as dimers, using the yeast-two-hybrid method, at different time points in evolution. One striking observation was that the gamma whole genome triplication at the origin of core eudicots induced strong rewiring of this dimeric MADS-domain protein interaction network^37^. While this major event also added many new interactions, the density of the network, i.e. the ratio of observed interactions and potential interactions, did not significantly change during evolution. This suggested that the relative number of actual protein-protein interactions in these networks was saturated (in term of network density). However, storage of more information could have become possible through higher order complex formation, mediated by the glue property of SEP3.

To have a better understanding of the origin and evolution of the glue capacity of SEP3 to mediate protein-protein interactions between other MADS-domain proteins, we reconstructed two yeast-three-hybrid (Y3H) protein interaction networks (PIN) at different time points (Supp. Fig. 1). A first network was reconstructed at 120 mya (million years ago), at the origin of eudicots before the gamma whole genome triplication named pre-PIN and a second at 109 mya at the split of the Asterids and Rosids, named post-gamma PIN or post-PIN. In addition, we applied the same methodology to investigate the extant protein networks of *Arabidopsis thaliana* (Ara-PIN) and *Solanum lycopersicum* (Sol-PIN)^37^. The interactions were quantitatively determined and the two-hybrid network was subtracted. We therefore defined a positive three-hybrid interaction as an interaction that was not present in two-hybrid or was significantly enhanced by the presence of SEP3 using the β-galactosidase assay (For Miller units of each interaction see Supp. Table 1).

**Figure 2:**
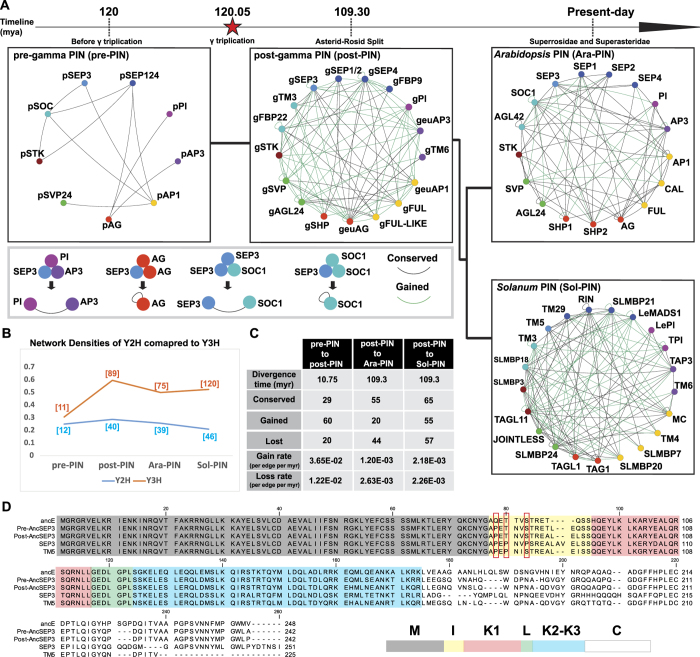
Evolution of SEP3 as a network hub. (**A**) Yeast three-hybrid (Y3H) assays of reconstructed ancestral and extant networks with (pSEP3, gSEP3, SEP3 and TM5) mediating ternary complexes formation. Black and green lines indicate conserved and novel gained ternary interactions respectively through comparison from pre-gamma PIN to post-gamma PIN and from post-gamma PIN to AraPIN and to Sol-PIN. An example is presented in the gray box where PI-AP3 and SEP3-SOC1 interactions are mediated by SEP3, which is represented by a line in network, and where AG-AG and SOC1-SOC1 interactions are mediated by SEP3, which is represented by a looped line in network. **(B)** Increase of network densities in yeast-three hybrid compared to yeast-two hybrid networks, total number of edges is shown above the graph line (orange: Y3H, blue: Y2H). **(C)** Total amount of conserved, gained and lost interactions in addition to the gain and loss rate (per edge per mya) between networks (gain and loss rate was defined as gained or lost interactions divided by number of potential interactions in networks times the divergence time). **(D)** Multiple sequence alignment of SEP3 and its resurrected ancestors in addition to its homolog in tomato TM5. Red boxes indicate proline accumulation in the I-domain. For Miller units of each interaction see Supp. Table.1.

Our results show that after the gamma triplication many more interactions were mediated by SEP3 and the network became denser (Fig. 2A,B), To compare pre-PIN to post-PIN, which have different sizes, the pre-PIN was triplicated (all interaction conserved), then the redundant nodes were removed, resulting in a transition network with the same network size as post-PIN. This increased density persisted after this major event, suggesting that although dimeric interactions in the network were reaching saturation, higher order complex formation provided a way to incorporate more novel interactions (Fig. 2B). Most novel SEP3-mediated interactions were gained between pre-PIN to post-PIN (Fig. 2C) where SEP3 strongly gained importance as a higher order complex glue protein through the gamma triplication and retained this function from then onwards^37^. The gain rate was balanced by the loss rate later in evolution and the overall network densities remained similar from post-PIN to Ara-PIN and Sol-PIN. This suggests that after the gamma triplication, a certain degree of complexity was reached in the SEP3-mediated network (possibly with a functional advantage) after which the network stabilizes and rewires rather than expands.

### SEP3 evolved from promiscuity to specificity

**Figure 3:**
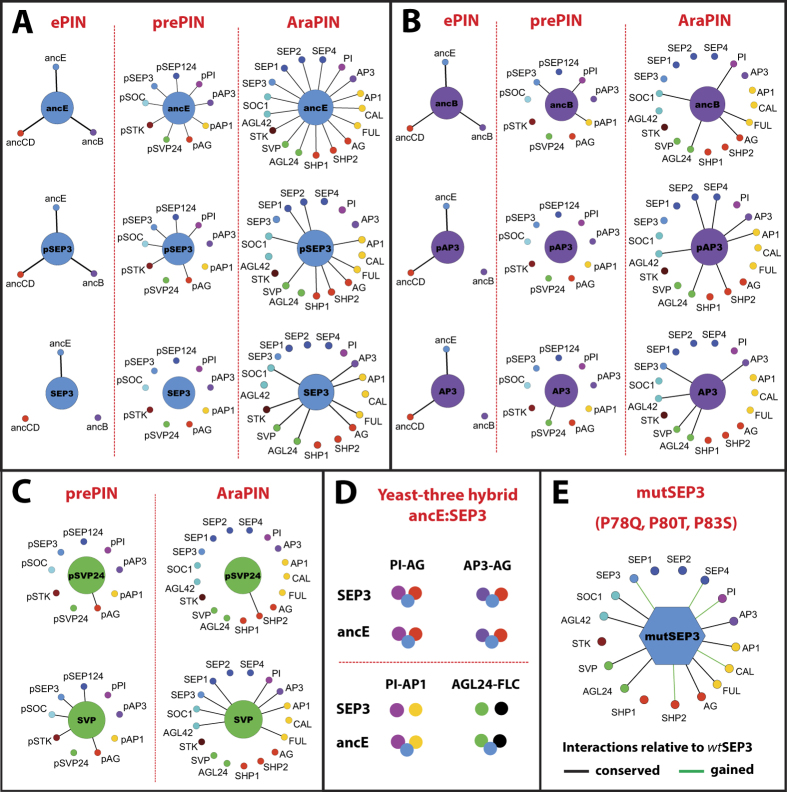
Yeast two-hybrid assays assessing the interaction of proteins in a network of a different age (swapping between networks) (**A**) SEP3, pSEP3 and ancE. (**B**) AP3, pAP3 and ancB. (**C**) SVP, pSVP24. (**D**) Three-hybrid interaction mediated by SEP3 vs ancE. (**E**) Two-hybrid assay results of mutant SEP3 (P78Q, P80T, P83S), showing the gain of total protein-protein interactions (green) from (8 to 13). For Miller units of each interaction see supp. Table. 2.

From the comparison of successive ancestral and extant protein-protein and SEP3-mediated protein-protein interaction networks, it becomes evident that after the gamma triplication the MADS-box interaction network underwent substantial rewiring resulting in novel dimeric and higher order complexes. We wanted to understand to which proteins in the network this rewiring can be attributed. In the case of the hub protein SEP3: the hub can evolve to affect many interactions, the other proteins in the network of SEP3 can evolve or both the hub and the other proteins can co-evolve. We selected two additional cases for comparison. One is the B lineage protein APETALA3 (AP3), which maintains few interactions and therefore can be considered an island and the other is SHORT VEGETATIVE PHASE (SVP), an intermediate between a hub and an island^37^. To investigate whether the protein or its partners evolved, we performed a reciprocal swapping experiment in which the interaction pattern of the ancestral and extant proteins from pre-PIN and Ara-PIN, in addition to an older network at 180 mya, at the origin of angiosperms, before the whole genome duplication event (epsilon) named ePIN^47^, were evaluated. More specifically, SEP3, AP3 and SVP from *Arabidopsis* were placed in ePIN and pre-PIN networks, pSEP3, pAP3 and pSVP24 ancestors were placed in the more ancient ePIN and the extant Ara-PIN network and finally the ancestor of SEP3 in the ePIN network (ancE) and AP3 in the ePIN network (ancB), were placed in the more recent pre-PIN and Ara-PIN networks (Fig. 3A–C). The most striking observation was that ancE, the oldest ancestral SEP3 protein, can bind to all MADS-domain proteins in all evolutionary networks. This indicates it acted as a promiscuous hub with low specificity (Fig. 3A). Although pSEP3 already lost some of its promiscuous binding ability to form dimers and SEP3 engages in the fewest protein interactions, both proteins still represent hubs in their respective networks yet with more specificity. This indicates that specificity of SEP3 was gained throughout the evolution of SEP3 and it evolved from a promiscuous hub to a specific hub. It is worth mentioning that ancE seems to have undergone subfunctionalization, since the total number of ancE interactions in pre-PIN is 9 which is equal to the sum of pSEP3 and pSEP124 interactions (6 + 3) respectively, and in Ara-PIN ancE has 17 interactions while SEP1,2,3 and 4 has 14 interactions (8 + 4 + 2 + 0) respectively^37^. This is a good example of protein subfunctionalization through evolution as an adaptation to increase in network complexity, and as a means of gaining interaction specificity.

The fact that the anceE protein was able to bind to every other protein in each network suggests that the hub property of ancE can be attributed to itself, rather than to its interaction partners. ancE encodes this property because when placed in more recent networks with different interaction partners, this promiscuity is retained. It therefore must be embedded in the tertiary structure of the protein. To control for the idea that all ancestral proteins have this same property, possibly because their structure can fold into multiple conformations, we verified whether also ancB and to a lesser extent, pSVP24 or pAP3 could interact promiscuously. We observed that ancB was only able to interact with five *Arabidopsis* MADS-domain proteins, similar to pAP3 (seven weak interactions) and AP3 (five interactions). pSVP24 interacted with only one other protein in both pre-PIN and Ara-PIN networks (Fig. 3B top, 3C top). This shows that promiscuity as in ancE is not universal among ancestral proteins. To further verify the functionality of the ancE protein, we tested whether it was able to mediate higher complex formation in a three-hybrid assay, like its descendant protein SEP3. We tested two known interactions that cannot form dimers in two-hybrid and require SEP3 to mediate the interaction in three-hybrid but now used ancE as the interaction mediator (Fig. 3D). In addition, we tested two interactions SEP3 cannot mediate. Our results showed that ancE was able to mediate both types of interactions. More broadly interpreted, if SEP3 is the “glue” of MADS-domain proteins^43^, ancE could be the “super-glue”.

### SEP3 lost conformational flexibility by acquiring prolines in the I domain

Because it is plausible that promiscuity in the SEP3 lineage can be attributed to SEP3, rather than to its interaction partners, we wanted to understand how the evolution of promiscuity to specificity occurred and which structural changes explain this. The fact that ancE was able to bind to all proteins in every network makes it more likely that an overall tertiary structure property such as disordered flexible regions was involved and not a specific domain-domain interaction. The folding of monomer proteins is a highly dynamic process, that involves the equilibrium between several conformational states. Once an interaction partner comes close enough, some of these conformations will fold into the dimer conformation, which represents a lower -more favourable- energy state, and the more flexible the monomer is the larger the difference between free and bound conformations^48^,^49^,^50^.

We noticed that the M-domain of SEP3 did not change at all throughout its evolution (Fig. 2D), indicating that residues in the M-domain which contributes to DNA binding specificity are under strong purifying selection. Substitutions were mainly in the I, K and C domains. For these regions, we focused on the linker regions between more structured domains, the I-domain and K1-K2 loop region, where a mutation might have a greater effect on the overall conformational dynamics by orienting the more structured domains. Proline residues characteristically increase the backbone rigidity due to their alpha-amino group bond to the side chain and we noticed the accumulation of proline residues in the I-domain in the SEP3 lineage. To evaluate the role of flexibility of the SEP3 I-domain for protein interaction, we back-substituted the proline residues 78, 80 and 83 in *Arabidopsis* SEP3 into the amino acid states present in ancE to Q T S respectively using site-directed mutagenesis. We hypothesized that substituting these residues would release the physical constraints on the I-domain folding and give it more flexibility, which would increase the conformational space and therefore increase the number of interactions. We indeed observed an increase in the number of interactions from 8 to 13 out of 17 interactions tested (Fig. 3E). To further investigate the link to flexibility *in silico*, we studied two peptides representing the I-domain of extant SEP3 (NYGAPEPNVPS), and the promiscuous-hub ancE (NYGAQETNVSS) for the number of low energy local minima conformations they can assume and we estimated their conformational entropies as flexibility descriptors. As expected, the SEP3 I-domain had the smallest conformational space (number of low energy local minima: 269) and the lowest conformational entropy (2.90 cal/K). The I-domain of ancE showed around 2.5 fold increase in conformational space (number of low energy local minima: 645) and higher conformational entropy (3.72 cal/K), which correlated with the number of interactions observed in our two-hybrid assay. The lowest energy state of ancE might be close to SEP3 state, but it is possible that the energy barriers between the states that bind other partners are lower compared to SEP3, allowing for fast inter conversion between the various binding conformations.

This accumulation of proline residues was observed in E-lineage proteins along their evolution, in *Arabidopsis* SEP3 and its paralogs SEP1 and SEP2, which can complement the *sep3* mutant^51^, as well as in the tomato SEP3 homolog TM5. As these prolines are not ancestral (not present in ancE), this suggests a similar molecular evolutionary strategy being utilized to reduce the hub property of these homologous and redundant hubs. Proline residues were also present in linker regions of other clades of MADS-domain proteins, and this seems to be a general evolutionary strategy of reducing flexibility in linker regions which affects the specificity of protein-protein interactions (Supp. Fig. 2). Together, these findings highlight the importance of structurally disordered domains in MADS-domain proteins, and the role of prolines in adjusting this flexibility between different domains, which is consistent with what has been observed for other hub proteins^52^,^53^,^54^.

### Large conformational changes between bound and free SEP3 K-domains

**Figure 4:**
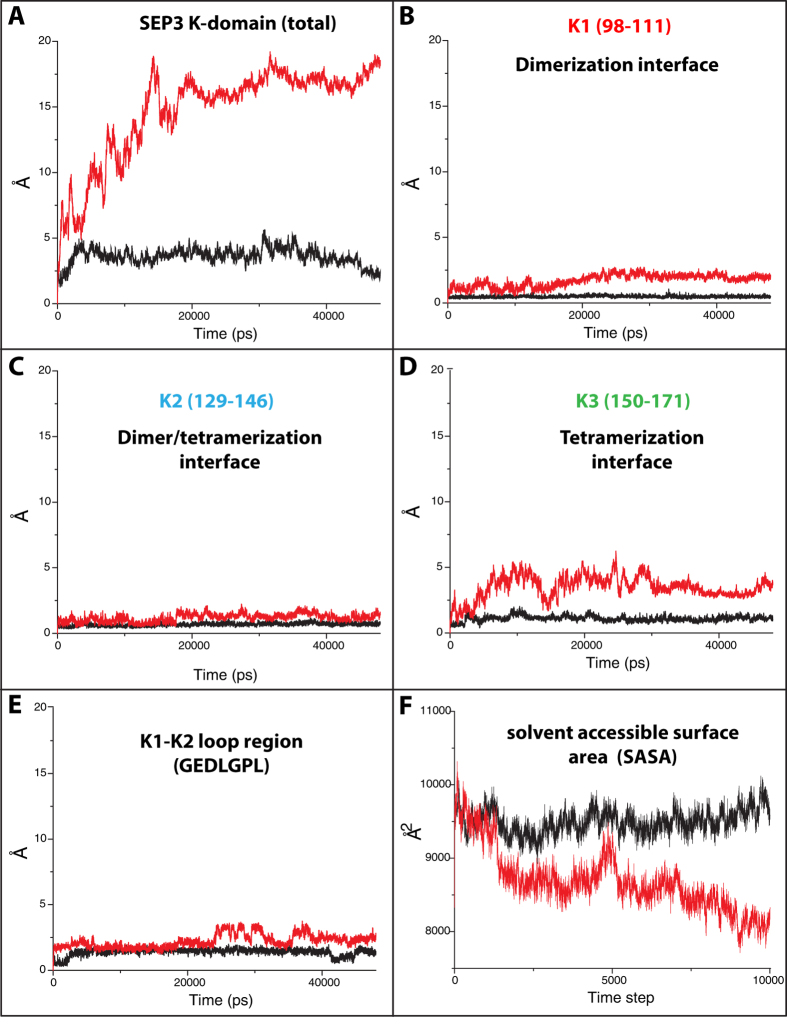
Molecular dynamics simulations of SEP3 K-domain tetramer (black lines) and monomer (red lines). (**A**) Complete K-domain RMSD calculations. **(B)** RMSD calculations for K1 subdomain residues 98–111. **(C)** RMSD calculations for K2 subdomain residues 129–146. (**D**) RMSD calculations for K3 subdomain residues 150–1171. **(E)** RMSD calculations for loop region between K1-K2 subdomains residues 117–123. **(F)** Solvent accessible surface area (SASA) calculations for one SEP3 K-domain in bound -tetramer complex- (black line) and free monomer (red line).

We noticed that the flexibility of SEP3 plays an important role in its capacity to bind to different partners in the network so that it can function as a hub. Protein crystal structures usually do not give a complete picture regarding flexibility and conformational changes in solution, like a cellular environment. Such information can be predicted using molecular dynamics simulations (MDS), where protein atoms are allowed to move dynamically under certain physicochemical conditions. Utilizing the recently published SEP3 K-domain crystal structure^44^, we carried out two MDS runs, one for the homo tetramer complex and one run for a monomer. We followed the movement of Cα atoms of the protein backbone during the simulation time (48 ns), and the root mean square deviation (RMSD) was reported as a function of time (Fig. 4A–E). One clear observation was that, once the tetramer complex is formed, it is quite stable, while the monomer, as expected, shows a large deviation from the dimer/tetramer conformation in the crystal structure (Fig. 4A). This points to a large conformational change induced upon binding. To understand this in more detail, we monitored the RMSD of individual subdomains. K1 residues (98–111) were previously reported to be involved in dimerization through a coiled-coil interaction. In the monomer simulation, the α-helix of the K1 subdomain was deformed, while the structure was more stable in the tetramer complex (Fig. 4B). The K2 and K3 subdomains **α**-helices formed a single continuous **α**-helix in the crystal structure, the K2 α-helix showed stability both in the monomer and in the tetrameric complex (Fig. 4C). The K3 α-helix, which was reported to be involved in tetramerization, was unstable as a monomer and deviated from the tetramer conformation (Fig. 4D), again pointing to an induced and stabilized conformation upon binding. The loop region between K1 and K2 subdomain residues (117–123) was predicted to lower the energy needed for dimers to bind, due to the presence of a proline residue, which helps to establish the 90° angle between K1 and K2 α-helices and expose the hydrophobic surface^55^. Also this region showed a small deviation during the simulation between bound and free K-domains (Fig. 4E). Finally, we monitored the solvent accessible surface area (SASA) of one bound (in tetramer) and one free SEP3 K-domain. Again the tetramer showed a stable conformation with a more open exposed surface, while the monomer folded on itself, which reduces the solvent accessible surface area (Fig. 4F). These large conformational changes between bound and free states support the importance of protein dynamics in oligomerization (Supp. Fig. 4), and it strengthens our previously mentioned results regarding the flexibility of SEP3, and probably other MADS-proteins in the network.

### SEP3 K domain loop conformational dynamics fine tune its hub property

Based on protein sequence alignments, the seven amino-acid loop between the K1 and K2 subdomains, seems to be a conserved feature in all type II MADS-domain proteins (Suppl. Fig. 2) and therefore it is likely important for their function. In wild-type SEP3, this loop has sequence GEDLGPL residues 117–123, and it was previously suggested^55^ that the presence of glycine-proline residues in class E SEP proteins forces the K1 and K2 subdomain α-helices apart to expose the hydrophobic residues, making dimerization more entropically favourable and contributing to the hub property of these proteins. Here we wanted to investigate this conserved region for its importance for protein interactions in our yeast two-hybrid networks.

To better understand its role in protein interaction, we first investigated the conformations the loop takes in bound versus unbound states. In the bound state, the last four residues, LGPL form a type IV β-turn (**φ**i + 1 = −50.ψi + 1 = −51, **φ**i + 2 = −62, ψi + 2 = −20) with a hydrogen bond between i and i + 3 of 2.34 Å. The angles in this turn, though it has a proline with cis configuration at i + 2, are different from regular type VIa1, VIa2 and VIb β-turns^56^,^57^. This turn structure contributes to the stability of the bound conformation. The first three more conserved residues in the loop, GED have no clear turn structure, but a salt bridge connects D119 (2.16 Å) and to a lesser extent E118 (3.68 Å) with R113 in the K1 subdomain. When the bound state switches to the monomeric unbound state in the molecular dynamics run, the loop structure goes through hydrogen bond rearrangement and loses the (LGPL) β-turn. Instead, two alternative turns are formed, a β-turn (GEDL) and pseudo-γ-turn (GPL). The salt-bridges between D119-R113 and E118-R113 remain present in both bound and unbound conformations.

**Figure 5:**
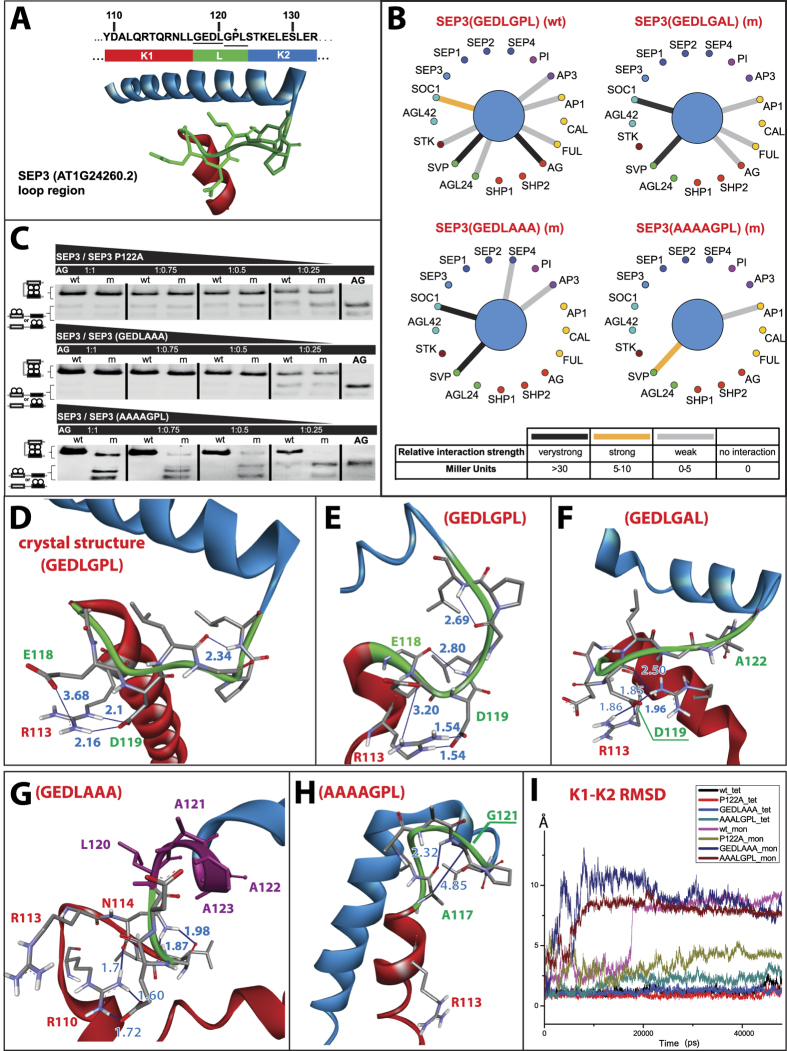
The role of SEP3 loop structure on PPI: (**A**) SEP3 loop sequence (GEDLGPL 117–123) located between the end of K1-subdomain and the beginning of K2-subdomain, underlined residues indicate 4Ala and 3Ala mutations while the star shows P122, and K-subdomain partial Crystal structure^44^. **(B)** Y2H assay for native SEP3 and three SEP3 mutants (SEP3(P122A), SEP3(G121A/P122A/L123A) and SEP3(G117A/D118A/E119A/L120A)). Protein interactions were shown by the straight lines. Relative interaction strength was illustrated by different colors (black, orange and grey) based on the values of Miller Units for all interactions. wt: wild type; m: mutant. **(C)** EMSA results show the binding affinities of native SEP3 and three SEP3 mutants with AG by SEP3 and SEP3 mutants titrations. Black triangles represent the concentration gradient of SEP3 and SEP3 mutants from highest to lowest comparing with constant concentration of AG in each group. The concentration ratios between AG and SEP3 or SEP3 mutants were demonstrated inside the black bars (1:1, 1:0.75, 1:0.50 and 1:0.25). Cartoons on the left side along the bands show the supposed quartet complex forming (top) and the dimerized forming (bottom) binding on the probe. The probe was a SEP3 promoter fragment containing two CArG boxes (see Methods) which was illustrated as double straight or bend lines harboring white and black bars in cartoons. **(D)** Loop dynamics, loop region in dimer conformation in resolved crystal structure (PDB: 4Ox0). And at 48 ns of MDS run of, **(E)** Native SEP3 K-domain **(F)** P122A mutant, **(G)** GEDLAAA mutant, **(H)** AAAAGPL mutant. Color codes (blue: K2 subdomain, red: K1 subdomain, green: loop region, magenta: K2 extended in GEDLAAA mutant, dark blue lines: represent possible hydrogen or polar bonds numbers next to it shows distance in angstrom). **(I)** RMSD of K1 residues 98–111 and K2 residues 129–146 as function of MDS time of native and loop mutants in monomer and tetramer states.

To understand the contribution of these loop structure conformations to the protein interaction specificity of SEP3, we introduced a number of mutations in the loop through site directed mutagenesis (Fig. 5A,B). We observed that these mutant proteins cannot take on the energetically more favourable conformation of the bound wild-type loop. A single P122A mutation resulted in 50% loss of protein interactions in yeast-two-hybrid. Because of its position, it is unlikely that proline 122 would be directly involved in protein interaction through intermolecular residue-residue contacts, but it can have an effect on the dynamics of dimerization. In this mutant, the β-turn (LGPL) was not present and no other β-turns were observed. The loop was more open and extended, placing the K1 and K2 subdomains further apart. The D119-R113 interaction was present and a new D119-R110 interaction formed (Fig. 5F). Therefore, the loss of interactions in the P122A mutant can be explained by the absence of the β-turn and the more extended loop structure, while the salt bridges help in maintaining the remaining interactions by keeping the K1-K2 subdomains away from each other, which is required for proper dimerization as seen in the crystal structure. The second mutant constituted of three alanine substitutions resulting in a GEDLAAA loop sequence. Again, about half of protein interactions were lost. The alanine residues formed an extension of the α-helix of the K2 subdomain, the β-turn was absent, D119-R113 was absent and a new D119-R110 had replaced it (Fig. 5G). This again suggests that the loss of protein interactions was due to the absence of the β-turn and the retention of interactions can again be attributed to D119-R110.

The third mutant with sequence AAAAGPL resulted in 75% loss of protein interactions and loss of all strong interactions (in Miller units) in comparison to the other two mutants. Interestingly, here the β-turn (LGPL) was rearranged into a pseudo-α-turn (AAAAG: **φ**i + 1 = −89, ψi + 1 = 10, **φ**i + 2 = −80, ψi + 2 = −55, **φ**i + 3 = −151, ψi + 3 = 30). The D119-R113 interaction was lost and it was not replaced with R110. A large hydrophobic surface was formed between the K1 and K2 subdomains. The RMSD calculations of K1 residues 98–111 and K2 residues 129–146 in the molecular dynamics simulations (Fig. 5I) show that the wild type SEP3 K-domain monomer escapes the dimer/tetramer conformation more slowly than the mutants, suggesting a relative stability of its bound conformation. The stability of these two helices and their relative orientation is largely altered in GEDLAAA and AAAAGPL mutants. Although the GEDLGAL mutant showed less deformation, it still deviated from both wild type monomer and tetramer conformations (for more details see Supp. Fig. 3).

The SEP3 mutants behaved somewhat differently in electrophoretic mobility shift assays (EMSA) with AGAMOUS, which allowed to test the interaction on DNA (Fig. 5C). At higher protein concentrations such as in EMSA, the effect of the mutations was reduced, and dimer/tetramer complexes still formed. Only the AAAAGPL mutant showed a very poor (only at high concentration) hetero tetramer formation with AG. The effect of high concentration is similar to what we observe in the crystal structure of SEP3, where homo dimer/tetramers were able to form, though SEP3 does not form homo dimer/tetramer in yeast-two and yeast-three hybrid assays (It could be that the interaction is too weak to be detected).

Together, we can conclude from these observations that the loop structure contributes to allow the SEP3 K-domain to take on its energetically most favourable conformation for binding. The presence of positively charged residues at the end of the K1 domain (4 residues away from the loop region) contributes to dimer formation through binding to a negatively charged residue within the loop. This position indeed is very conserved (94% R and 6% K, Supp. Fig. 2). The rigidity of the loop region and the involvement of proline 122 seems not to be the main factor determining SEP3 its ability to dimerize. Even the wild type SEP3 loop monomer goes through conformational changes and molecular dynamics results show some conformational stability after 20 ns of simulation time. Indeed, the P122A mutant is still able to interact in Y2H and EMSA assays, but less favourably. Also other, non E class proteins like CAULIFLOWER (CAL), have a proline residue at the same position but has only one interaction in the two-hybrid assay and its homolog FRUITFULL (FUL), which does not have a proline in its loop region, has 9 interactions in yeast-two hybrid assays^37^. Overall, these results point to the combined importance of conformational dynamics for binding in addition to residue-residue contacts on the interaction surface between the proteins. This has to be taken into account when addressing questions such as what makes certain proteins hubs and others isolated islands.

### Not all MADS-domain proteins are equal

**Figure 6:**
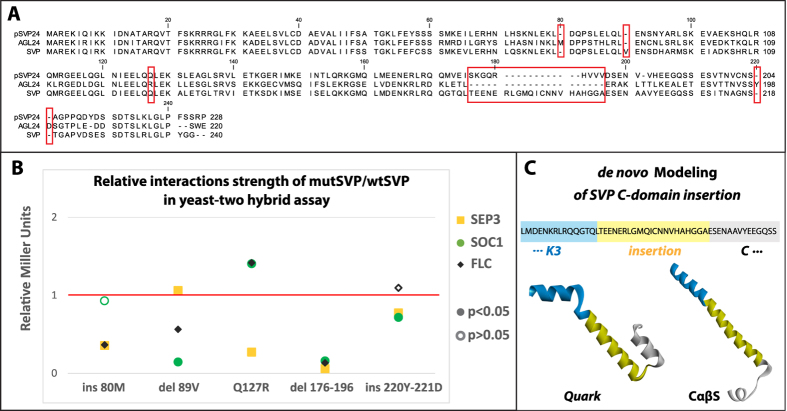
SVP site-directed mutagenesis Y2H assay, and C-domain insertion *de novo* modeling. (**A**) Multiple sequence alignment of pSVP24, SVP and AGL24, red squares indicate mutation sites. (**B**) Mutant SVP two-hybrid relative to *wt*SVP two-hybrid (red base line 1 = 100%). (**C**) Top *de novo* models of SVP C-domain insertion using Quark and CαβS servers.

In the case of the SEP3 clade, the number of protein interactions may have been controlled through adjusting the dynamics of protein folding by accumulating proline residues in the linker regions between different domains. A question is now whether this strategy was also utilized in a different clade, for instance SVP and whether alternative processes exist. From the multiple sequence alignment of SVP, AGL24 and their common ancestor pSVP24 (Fig. 6A), we noticed the presence of proline residues in the I-domain in all three proteins. However, pSVP24 has only one protein interaction in pre-PIN and in Ara-PIN, while SVP has eight interactions in Ara-PIN and five interactions in pre-PIN, which is a number similar to the hub SEP3 in both networks respectively (see Fig. 3). If the same molecular evolutionary process was behind this gain of interactions, we would expect a substitution of the proline in the I-domain, but that was not the case. The main differences in the SVP I-domain were the insertion of a valine residue at position 89 and an insertion of a methionine at position 80 in AGL24. The second linker loop region between the K1 and K2 subdomains was also conserved. Yet, a prominent difference between SVP and AGL24 is an insertion in SVP of 21 unique amino acids at positions 176–196 (between K3 subdomain and the C-domain, Fig. 6C) with sequence TEENERLGMQICNNVHAHGGA. In addition, SVP has a small two amino acid deletions at 220–221 in the C-domain, Tyr-Glu. While there are more differences between SVP and AGL24, we chose to focus on the importance of the above mutations for their effect on interactions of SVP. To verify whether the mutations could explain the gain of interactions observed in SVP, we introduced them through site-directed mutagenesis. Each of the mutant SVP proteins was assayed for interaction with SEP3, SOC1 and FLC, which are known interaction partners of wild-type SVP in two-hybrid assays (Fig. 6B).

The behavior of the mutants varied greatly, starting with I-domain mutations. Del89V showed a minor (6%) but significant increase in interaction strength with SEP3, but decreased FLC binding by 44%, and SOC1 binding by 86%. While ins80M had almost no effect on SOC1 binding, it decreased binding of both FLC and SEP3 by ~64%. The K2-subdomain mutation Q127R decreased SEP3 binding by 73%, but increased binding to both FLC and SOC1 by ~40%. The C-domain insertion 220–221 YD had no significant effect on FLC binding, but decreased SEP3 and SOC1 binding by 22% and 28% respectively. This variation between different SVP heterodimers suggests that these mutations are on the protein-protein interaction interface, rather than being mutations that affect an overall tertiary structure fold as in the case of proline mutations in SEP3. By contrast, the deletion of the SVP unique C-domain insertion 176–196 had a global effect, reducing all binding to FLC, SEP3 and SOC1 by ~84–95%. This mutation was not expected to have such a strong effect, since the C-domain was thought not to play a significant role in dimerization, but rather to affect tertiary complex formation. The C-domain contains the activation domain for some of the MADS-domain transcription factors^58^, but in case of SVP this extra C-domain insertion is apparently essential for dimerization as well. Interestingly, it was the most prominent feature that distinguishes SVP from its ancestor pSVP24. De novo modeling using servers Quark^59^ and CABS-fold^60^,^61^ predicted an α-helix structure for this insertion (Fig. 6C). Since the main interaction interfaces in MADS-domain proteins are between α-helices, we can argue that the major gain of interactions observed in SVP is mainly due to an extension of the α-helix, through this unique extra C-domain insertion. This supports the idea that there are multiple evolutionary strategies being utilized during MADS-domain protein evolution. Also it points to different dimerization interfaces between MADS-domain proteins. An elongated dimer conformation was suggested^55^ for the AG homodimer where the loop between K1 and K2 subdomains form a 180° angle instead of 90°. While SEP3 does not require the C-domain, SVP does, and the proper folding to dimer conformation is highly affected by M, I and C domains, at least in case of SVP and SEP3.

## Discussion

In this study we investigated the molecular mechanisms and evolutionary trajectories behind the evolution of hub and non-hub proteins in the network of MADS-domain transcription factors. We noticed that when the density of dimeric interactions in the network reached saturation, the glue protein SEP3 had an important role in increasing network density. This persisted after the major whole genome duplication event gamma, suggesting that higher order complex formation provided a way to incorporate more novel interactions from the gamma triplication onwards. We highlighted the importance of structural flexibility in hub proteins, a property that allows them to adopt different structural conformations upon binding to targets (induced fit model), and/or by lowering the energy barrier between different the conformations of the free state proteins, allowing for a higher rate of inter conversion between conformations (conformational selection model), boosting their binding capacity, and keeping a degree of interaction promiscuity.

This structural flexibility seems to be a key hub property, not only in large hubs but in small hubs as well, rather than surface residues charges as a main contributor to their interaction capacity. We followed the evolutionary history of the hub protein SEP3 and found that its ancestor at 180 mya (ancE) had a very high promiscuity level and was able to bind to all other MADS-domain proteins in every protein network. This universal hub property was largely due to its structural flexibility, as it was lost through its evolution through accumulation of proline residues in linker regions between its subdomains. Also, ancE seems to have undergone subfunctionalization along its evolution, as the number of its interacting partners adds up approximately to the number of interacting partners of SEP1,2,3 and 4 in each network. This evolutionary strategy of having multiple specific hubs instead of super-hubs may provide a higher level of network robustness, since a failure in hubs with lower numbers of interactions will have less effect than a central hub failure. We also illustrated that the high number of interactions of hubs was determined mainly by the hubs themselves, rather than by their interaction partners.

It appears that specificity evolved through selection of conformational folding states by controlling the degree of disorder, followed by fine tuning through electrostatic/hydrophobic residue-residue interaction on the interaction interface. We also noticed the presence of proline residues in linker regions between protein domains (I-domain, K1-K2 loop region) in MADS proteins. This may have led to reduced flexibility along their evolution, an observation that seems to be an exception to what has been reported before^17^, where evolutionarily more recent subunits were generally more flexible than evolutionarily older subunits.

It is worth mentioning that yeast-two hybrid assays in general might increase the number of observed protein-protein interaction due to unnatural high expression level of the cloned genes, thus the *in vivo* total number of interactions could be lower than the yeast two/three hybrid reported interactions. But the trend of an increasing or decreasing number of interacting partners would not be affected.

Dimerization in MADS-domain proteins requires proper folding at the event. The molecular dynamic simulation showed that this fold is induced (dynamic) rather than *a priori* existing in the monomer state (induced fit). After allowing the crystal structure to relax we observed rearrangements in the loop region in addition to the α-helices of K1, K2 and K3 subdomains. The wild type SEP3 escaped the dimer conformation more slowly than the mutants. The second case we studied was SVP, which evolved from not being a hub and gained more protein interactions in evolution. We illustrated that it used a different molecular strategy during its evolution. An additional α-helix C-terminal insertion was gained through its evolution, increasing the number of protein interactions with other MADS-domain proteins in the network. Surprisingly this extra helix seems to be crucial for heterodimerization, since the deletion mutants of this unique 21 residues lost their capacity to dimerize through MIK-C^mut^ domains, while SEP3 dimerization does not require the C-domain^44^,^62^. Therefore previously reported MADS-domain proteins with altered C-domains^63^,^64^, which resulted in neo-functionalization might be functionally explained by protein-protein interaction (dimerization in addition to tetramerization). This adds to the idea that protein-protein interaction is a complex process. At least in networks of MADS-domain proteins, each couple may have a unique mode of dimerization and each member its ability to interact may have evolved uniquely, even though MADS-domain proteins have high sequence similarity. Resolving full length 3D structures of different homo/hetero-dimers/tetramers of this family will help to further advance our understanding of such complex molecular mechanisms and of protein-protein interaction network evolution.

## Methods

### Ancestral gene reconstruction of different MADS-box protein networks

Ancestral genes from ePIN were used from Ruelens *et al*. (unpublished)^47^. Ancestral genes from pre-PIN and post-PIN were used from Zhang *et al*. (unpublished data)^37^. Protein sequences were obtained from available databases Genbank, OneKP, Phytozome, or from the *Gunnera maniacata* and *Pachysandra terminalis* RNAseq dataset from (Vekemans, D. *et al*.)^28^. Sequences were initially aligned with MUSCLE^65^ and manually curated in McClade 4.08^66^. Phylogenies were constructed using PhyML 3.0 as implemented in Geneious 5.4 or by RAxML^67^,^68^,^69^ using the GTR substitution model. All phylogenies were manually improved up until the order level to match with the angiosperm phylogeny^70^,^71^. Branch lengths were estimated on these manually curated trees using RAxML 7.0.4^72^ with the JTT + G or JTT + I + G models of protein evolution, as determined by ProtTest 2.4^73^. The indel history of the sequence alignments was manually reconstructed. All insertions that occurred after the gamma-event were deleted from the matrix. Next, the nucleotide sequence alignments were translated to proteins. The optimized gene trees with branch lengths, the protein alignments and best-fit model of evolution were then used for maximum likelihood marginal reconstruction implemented in PAML4.4^74^. Ancestral sequences were estimated at the last node before the γ triplication (after the divergence of Buxales and before the divergence of Gunnerales) and at the Asterid-Rosid split. Finally, the obtained ancestral protein sequences were converted to nucleotide sequences, codon optimized for yeast *S.cerevisiae* and *A. thaliana* and synthesized by Genscript USA.

### Site directed mutagenesis

Mutations were introduced in the expression vectors through PCR using Phusion polymerase (initial denaturation 98 °C 3 min, denaturation 98 °C 60 s, annealing 55 °C 60 s, elongation 68 °C 30 s/kb of plasmid, 18 cycles) and the following primers:

SEP3 P78,80,83-QTS (TGGAGCACAAGAAACCAATGTGTCTTCAAGAGAGGCCT)

SEP3 P122A (GGAGAAGATCTTGGAGCGCTAAGTACAAAGGAG)

SEP3 GEDLAAA (GGAGAAGATCTTGCGGCGGCGAGTACAAAGGAG)

SEP3 AAAAGPL (CAAAGGAATCTGTTGGCGGCGGCGGCGGGACCTCTAAGTACAAAG)

SVP 80 M-insertion (AA AGAACTTGGAGAAGCTTATGGATCAGCCATCTCTTGAG)

SVP 89 V-deletion (ATCTCTTGAGTTACAGCTGGAGAACAGTGATCACGCC)

SVP 175–195 deletion (CAAGGAACGCAACTAGAATCGGAGAACGCTG)

SVP 220 Y-221 D insertion (CCGGAAACTCTTATGATACCGGAGCGCCTGTT)

SVP Q127R (GACATTGAAGAGCTTCAGAGACTAGAGAAGGCCCTTGAAACT)

After the PCR reaction, 1 μl DpnI enzyme (NEB) was added to each PCR reaction tube and incubated at 37 °C overnight, followed by column purification using Wizard^®^ SV Gel and PCR Clean-Up System from which 5ul was transformed in Top10 chemically competent cells. Successful mutagenesis was confirmed by sequencing.

### Yeast-two and yeast-three hybrid assays

For yeast two hybrid (Y2H), recombinant pGADT7 AD Vector and pGBKT7 BD vectors (Clontech) containing MADS-box genes, were co-transformed into Y187 yeast strain. Successful transformation was confirmed by selective growth on Synthetic Defined (SD) 2% agar plates lacking essential amino acids Leu and Trp. For yeast three hybrid (Y3H), to detect the ternary complex formation, a third recombinant pTFT1 vector^75^, containing SEP3 or an ancestor SEP3 (ancE/pSEP3/gSEP3) in *Arabidopsis thaliana* network or TM5 in *Solanum lycopersicum* network, was co-transformed in addition to the AD and BD vectors, the successful transformation was confirmed by selective growth on Synthetic Defined (SD) 2% agar plates lacking Adenine and essential amino acids Leu and Trp. All yeast transformations were carried out following the LiAc-mediated yeast transformation as described in Clontech Yeast Protocols Handbook. Auto-activation was tested through recombinant BD vector containing MADS-box gene, co-transformed with empty AD vector in Y2H, while in Y3H auto-activation was tested by, co-transformation of recombinant pTFT1 vector containing either SEP3 or an ancestral SEP3 in *Arabidopsis thaliana* networks, or TM5 in *Solanum lycopersicum* networks, and empty AD vector. Co-transformation of empty BD vector with empty AD vector in Y2H, and empty BD vector with empty AD vector and empty pTFT1 vector in Y3H, was used as internal reference control of reporter gene leakiness. To analyze the protein-protein interaction quantitatively, β-galactosidase activity was detected using ortho-Nitrophenyl-β-galactoside (ONPG) as a substrate^37^. Y2H reads were subtracted from Y3H results, to get the true positive interaction which has been enhanced via SEP3. Positive values represent gain/conserved interactions through Y3H, while negative values represent lost/false positive interactions.

### EMSA

A dsDNA oligonucleotide probe was cloned into pGEM-T vector (Promega). The DNA probe containing two CArG boxes, was derived from SEP3 promoter region in *Arabidopsis thaliana*^41^, the probe was amplified and double-labeled with 5′ end-biotin, using PCR with 5′ end-biotin labeled specific primers:

Forward: (5′ end-biotin-CATGGCCGCGGGATT-3′),

Reverse: (5′ end-biotin-GCGGCCGCACTAGTGATT-3′)

EMSA 5′ end-biotin double-labeled Oligonucleotide Probe Sequence (CArG-boxes are underlined):

(CATGGCCGCGGGATTTTGACGATAACTCCATCTTTCTATTTTGGGTAACGAGGTCCCCTTCCCATTACGTCTTGACGTGGACCCTGTCCGTCTATTTTTAGCAGAATCACTAGTGCGGCCGC).

Then the probe was purified on 1% agarose gel (Promega). Mutant SEP3 genes generated in previously described site-directed mutagenesis step, in addition to AG gene, were amplified via PCR, then cloned downstream of an SP6 RNA polymerase promoter into a pSPUTK *in vitro* translation vector. Proteins synthesis was carried out using TNT^®^ SP6 High-Yield Wheat Germ Protein Expression System (Promega), into a total volume of 10 μl according to the ratio of the reaction components of the manufacturer. It contained a 6 μl TNT^®^ SP6 High-Yield Wheat Germ Master Mix and a total of 1 μg plasmid DNA in each tube. Reaction mix was incubated for 2 hours at 25 °C. The binding reaction mixt has total volume of 12 μl, containing 1.13 mM EDTA (pH 8.0), 0.024% BSA, 7.2 mM HEPES-NaOH (pH 7.3), 0.72 mM DTT, 57.72 μg/ml salmon sperm DNA, 1.27 mM spermidine, 2.5% CHAPS, 8% glycerol, 40 fmol double-labeled oligonucleotide probe and 2 μl *in vitro* expressed proteins, according to the previous described mixture^75^. The binding reaction was incubated on ice for 30 min and then loaded on a native 5% polyacrylamide gel. After 3 hours of gel running at 75 V, the gel was blot to the BrightStar^®^-Plus positively charged nylon membrane (Ambion^®^) overnight. The DNA band shift was detected using chemiluminescent nucleic acid detection module (Thermo Scientific).

### Homology modeling

SEP3 M-domain was homology modeled based on available (SRF, MEF2) crystal structures, two separate models were obtained for SEP3 M-domain using (SWISS-MODEL Server)^76^,^77^,^78^,^79^. The I-domain was added as an extended amino acids, followed by superpositioning the K-domain 4OX0 to the I-domain manually, structures were refined via (GalaxyWEB Server:GalaxyRefine method)^80^,^81^, then structures were docked using (SwarmDock Server)^82^,^83^,^84^,^85^ and refined again via GalaxyWEB Server (GalaxyRefine method)^80^,^81^.

### *In silico* Loop mutagenesis

SEP3 (GEDLGAL), SEP3 (AAAAGPL) and SEP3 (GEDLAAA) tetramer structures were modeled by manually mutating the residues of wtSEP tetramer experimental structure (PDB ID 4OX0)^44^, through the Maestro interface^86^. Mutated loops were refined through Prime software^87^ using the extended serial loop sampling method. VSGB^88^ was used as implicit solvation model. No constrain was applied and no limit was set to Cα movements. All side chains within 10 Å from the loop residues were optimized.

### Molecular dynamics simulations

Molecular dynamics simulations of WT SEP3, SEP3 (GEDLGAL), SEP3 (AAAAGPL) and SEP3 (GEDLAAA) were run in explicit solvent, using the TIP4P^89^ water model in a Periodic Boundary Conditions orthorhombic box. Analogous to recently published studies^90^,^91^, Desmond MD system^92^,^93^ was used to set up and run the MD simulations. The simulated environments were built using the system builder utility, with the structures being neutralized by Na^+^ and Cl^−^ ions, which were added until a concentration of 0.15 M was reached. A series of minimizations and short MD simulations were carried out to relax the model systems, by means of a relaxation protocol consisting of six stages: (i) minimization with the solute restrained; (ii) minimization without restraints; (iii) simulation (12 ps) in the NVT ensemble using a Berendsen thermostat (10 K) with non-hydrogen solute atoms restrained; (iv) simulation (12 ps) in the NPT ensemble using a Berendsen thermostat (10 K) and a Berendsen barostat (1 atm) with non-hydrogen solute atoms restrained; (v) simulation (24 ps) in the NPT ensemble using a Berendsen thermostat (300 K) and a Berendsen barostat (1 atm) with non-hydrogen solute atoms restrained; (vi) unrestrained simulation (24 ps) in the NPT ensemble using a Berendsen thermostat (300 K) and a Berendsen barostat (1 atm). At this point, 48 ns long MD simulations were carried out at a temperature of 300° K in the NPT ensemble using a Nose-Hoover chain thermostat and a Martyna-Tobias-Klein barostat (1.01325 bar). Recording intervals for trajectories was set to 4.8 ns. Trajectories were analyzed using Desmond Simulation Event Analysis^94^ and VMD^95^.

### Conformational searches and conformational entropy prediction

NYGAPEPNVPS and NYGAQETNVSS peotides were investigated for their conformational behavior in solution using the MacroModel software^96^. The two peptides were sketched using the Maestro^86^ interface, capped with ACE and NMA residues, and were submitted to 500000 steps of mixed torsional/large-scale low mode sampling conformational search. GB/SA^97^ was used as implicit solvation model. Each generated conformation was minimized using a maximum of 2500 steps of Polak-Ribière conjugate gradient minimization, using a gradient threshold of 1.0 Kcal/mol as convergence criterion during the conformational search. A gradient threshold of 50 cal/mol was instead used as convergence criterion for the final minimization of the conformational sets. Assuming a Boltzmann distribution, probabilities for each conformer were calculated from potential energies. Conformational entropies were computed from conformer probabilities^98^,^99^ using the equation:


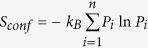


where k_B_ is the Boltzmann constant, and P_i_ is the probability of the i^th^ conformer.

## Additional Information

**How to cite this article:** Alhindi, T. *et al*. Protein interaction evolution from promiscuity to specificity with reduced flexibility in an increasingly complex network. *Sci. Rep.* **7**, 44948; doi: 10.1038/srep44948 (2017).

**Publisher's note:** Springer Nature remains neutral with regard to jurisdictional claims in published maps and institutional affiliations.

## Supplementary Material

Supplementary Information

## Figures and Tables

**Figure 1:**
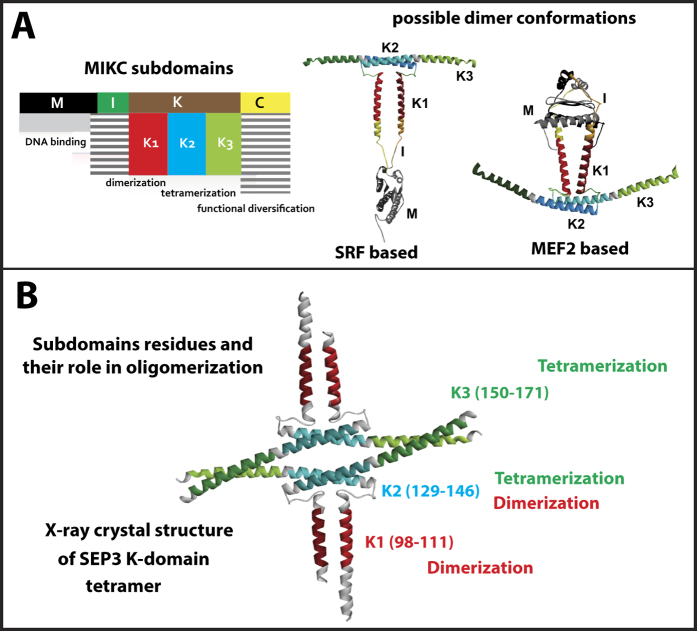
SEP3 structural information. (**A**) Left: MIKC-type subdomains and their reported functions, right: possible dimer conformations based on available MADS-domain protein 3D structure of SRF and MEF2 homology modeling. (**B**) Resolved X-ray crystal structure of the SEP3 K-domain, with its subdomain K1, K2 and K3 and their role in oligomerization^44^.
