# Supplementary material for: Protein interaction evolution from promiscuity to specificity with reduced flexibility in an increasingly complex network
Source: Sci Rep. 2017 Mar 24;7:44948. doi: 10.1038/srep44948 (PMC5364480; doi:10.1038/srep44948)
Supplement: Supplementary Information [file srep44948-s1.pdf]

Protein interaction evolution from promiscuity to specificity with reduced flexibility in an increasingly complex network

Al Hindi, T.[1], Zhang, Z.[1], Ruelens, P.[1], Coenen, H.[1], Degroote, H[1]. Iraci, N[2]. and Geuten, K[1,*].

[1]Department of Biology, KU Leuven, Leuven, Belgium.

[2]Department of Pharmacy, University of Salerno, Salerno, Italy

[*]Corresponding author: koen.geuten@kuleuven.be

Supplementary data and methods

Supp. Figure 1. Evolution of selected MADS-box proteins of interest. Simplified phylogenetic tree, the positions at which protein interaction networks were inferred are indicated (red stars). Pie charts represent the posterior probability of the inferred SEP ancestors gSEP3, pSEP3 and ancE.

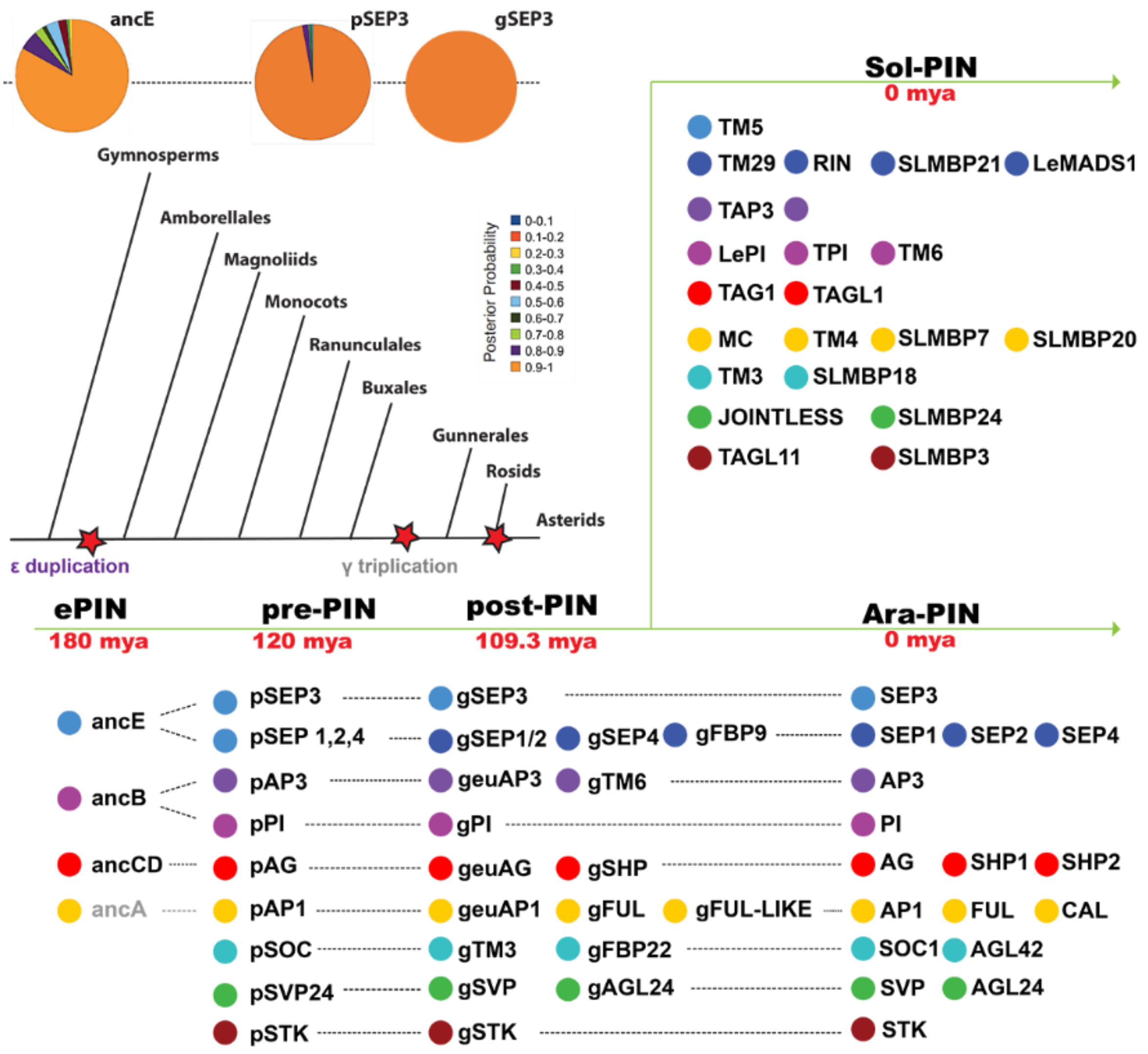

Supp. Figure 2. Presence of proline residues in I-domain and K1-K2 loop region of MADS-domain proteins. Proline residues are highlighted with red color, conservation of positively charged residues at i-4 from the loop region is highlighted with blue square.

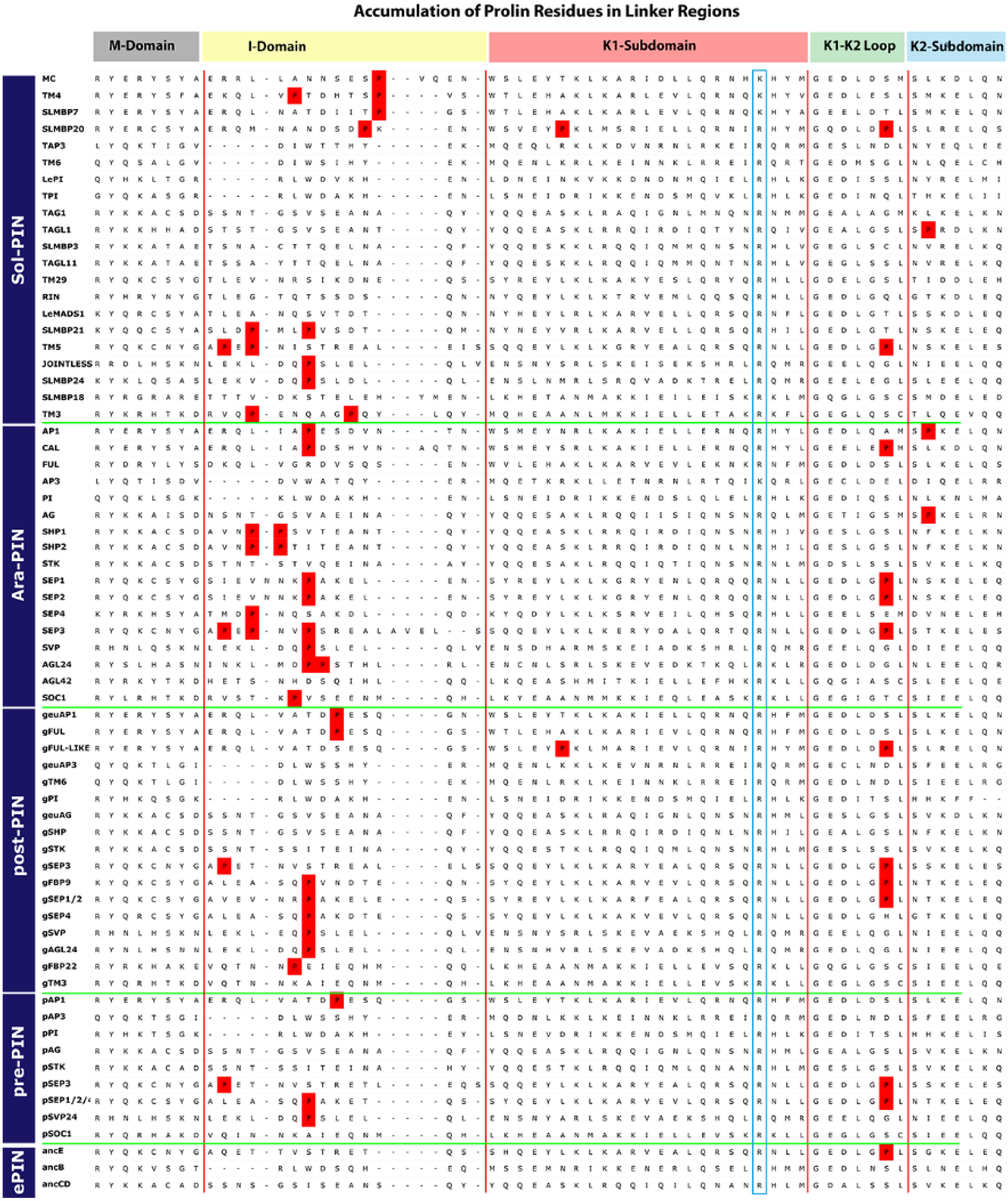

Supp. Figure 3. Detailed RMSD calculations of wtSEP3 and loop mutants.

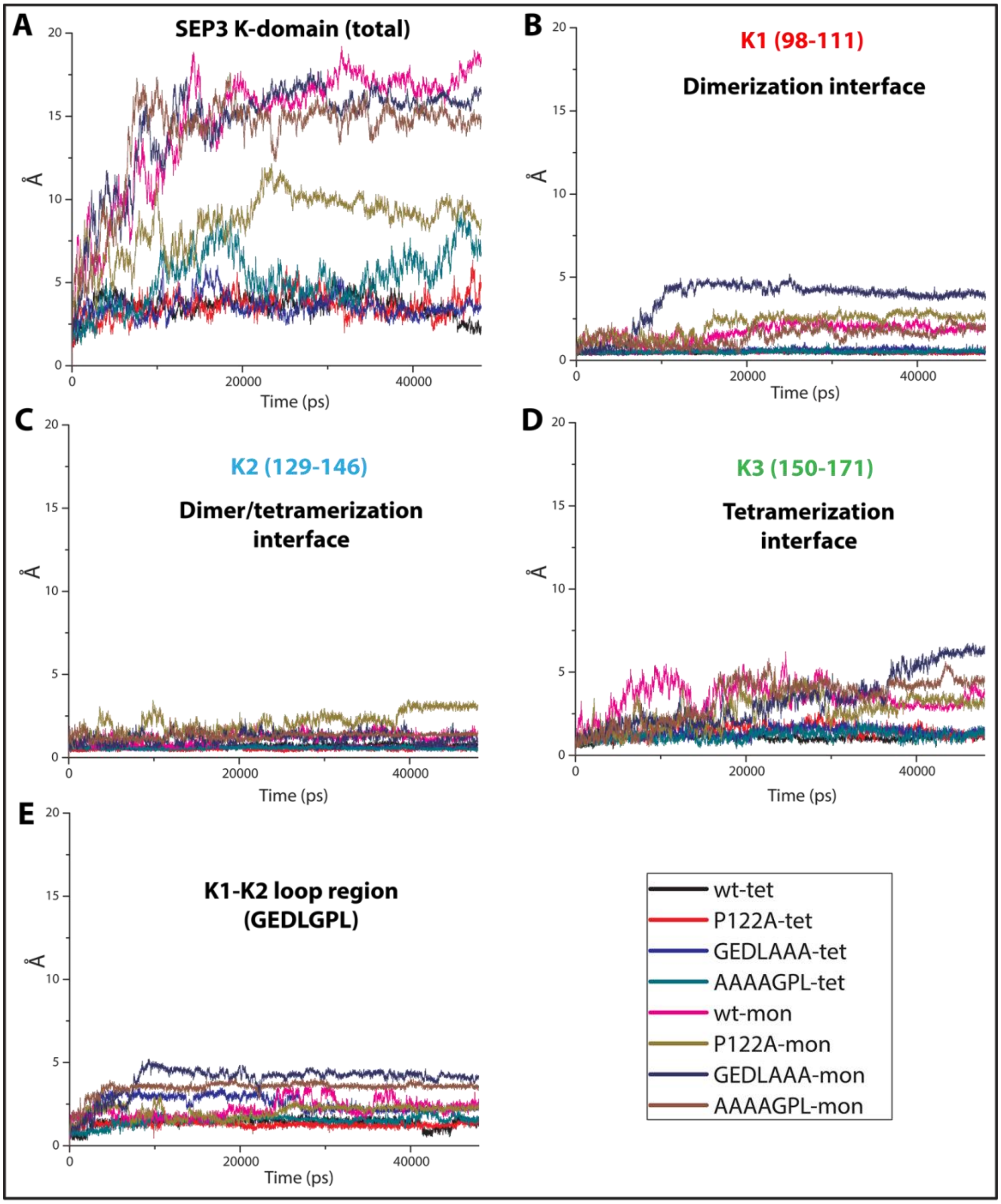

Supp. Figure 4. SEP3 K-domain tertiary structure after 40ns of MDS. Top: single K-domain from tetramer crystal structure, Middle: single K-domain from tetramer MDS at 40ns, Bottom: K-domain of monomer MDS at 40ns.

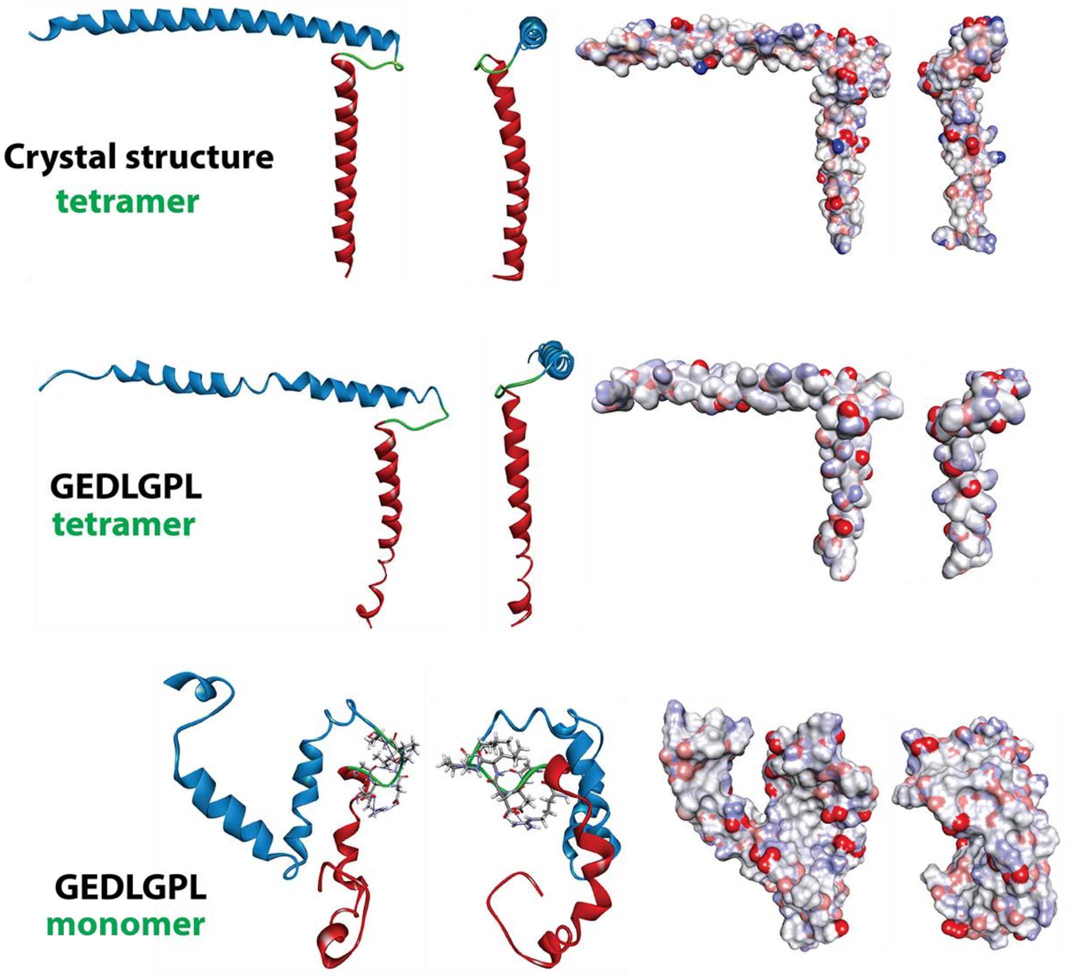

Supp. Table 1. Measurements of β-galactoside (ONPG) assays (miller units) of yeast-three hybrid assays.

Sol-PIN yeast-three hybrid

BD	pTFT	AD	Miller units
LeMADS1	TM5	LeMADS1	0.9148
SLMBP21	TM5	SLMBP21	2.4256
MC	TM5	MC	1.0182
TM3	TM5	TM3	3.1664
TAGL11	TM5	TAGL11	1.5665
TAG1	TM5	TAG1	1.2847
TAGL1	TM5	TAGL1	1.2225
TPI	TM5	TPI	0.4820
SLMBP24	TM5	SLMBP24	10.9563
TM5	TM5	SLMBP21	2.9820
TM29	TM5	TM5	1.5281
TM29	TM5	LeMADS1	1.9907
TM29	TM5	SLMBP3	3.7666
TM29	TM5	TAGL11	2.2679
TM29	TM5	SLMBP24	7.4417
RIN	TM5	TM5	1.9901
RIN	TM5	SLMBP24	7.9303
LeMADS1	TM5	SLMBP21	0.6582
LeMADS1	TM5	TM3	2.8612
LeMADS1	TM5	SLMBP3	1.8021
LeMADS1	TM5	SLMBP24	7.7505
SLMBP21	TM5	TM29	1.8690
SLMBP21	TM5	SLMBP18	1.8401
SLMBP21	TM5	SLMBP3	2.3352
SLMBP21	TM5	TAGL11	1.3326
MC	TM5	TM5	3.6702
MC	TM5	TM29	1.7458
MC	TM5	RIN	2.0395
MC	TM5	SLMBP21	2.3796
MC	TM5	SLMBP18	1.5317
MC	TM5	TM3	0.9770
MC	TM5	TAGL11	2.1614
MC	TM5	TAGL1	2.1705
MC	TM5	TM6	1.4480
MC	TM5	SLMBP24	1.7388
SLMBP20	TM5	RIN	1.5028
SLMBP20	TM5	LeMADS1	1.2910
SLMBP20	TM5	SLMBP3	1.3100
SLMBP20	TM5	TAGL11	1.8789
SLMBP20	TM5	TAGL1	1.1430
SLMBP20	TM5	SLMBP24	2.7011
TM4	TM5	RIN	1.3527
TM4	TM5	SLMBP21	1.1708
TM4	TM5	TAGL11	0.8928
SLMBP7	TM5	TM5	1.4863
SLMBP7	TM5	TM3	10.3416
SLMBP18	TM5	RIN	1.2239
SLMBP18	TM5	LeMADS1	2.4992
SLMBP18	TM5	SLMBP3	2.2277
SLMBP18	TM5	TAGL11	1.8088
SLMBP18	TM5	TAGL1	1.5717
SLMBP18	TM5	SLMBP24	2.3974
TM3	TM5	RIN	4.7024
TM3	TM5	SLMBP18	2.2349
TM3	TM5	SLMBP3	2.0225
TM3	TM5	TAGL11	2.0154
TAGL11	TM5	LeMADS1	1.4057
TAGL11	TM5	SLMBP3	3.6676
TAGL11	TM5	TAGL1	1.4385
TAGL11	TM5	JOINTLESS	3.2128
TAG1	TM5	TM29	1.8432
TAG1	TM5	SLMBP3	2.6453
TAG1	TM5	TAGL11	2.2005
TAG1	TM5	TAGL1	1.6834
TAG1	TM5	SLMBP24	2.5429
TAGL1	TM5	TM3	1.9340
TAGL1	TM5	SLMBP3	2.6181
LePI	TM5	TAGL11	1.7701
LePI	TM5	TAG1	5.0378
LePI	TM5	SLMBP24	1.3170
TPI	TM5	TM29	1.9925
TPI	TM5	LeMADS1	1.2618
TPI	TM5	SLMBP21	2.9862
TPI	TM5	TM3	1.4439
TPI	TM5	SLMBP3	2.9646
TPI	TM5	TAGL11	1.4652
TPI	TM5	TAGL1	1.0162
TPI	TM5	JOINTLESS	1.4739
TM6	TM5	TAGL11	1.0278
TM6	TM5	SLMBP24	2.3078
TAP3	TM5	TM5	1.6905
TAP3	TM5	TM29	0.9951
TAP3	TM5	LeMADS1	2.2092
TAP3	TM5	MC	1.4015
TAP3	TM5	SLMBP18	1.8294
TAP3	TM5	TAGL11	2.5341
TAP3	TM5	TAGL1	1.4905
TAP3	TM5	LePI	2.0685
TAP3	TM5	TPI	1.5160
TAP3	TM5	JOINTLESS	1.9184
TAP3	TM5	SLMBP24	2.3397
JOINTLESS	TM5	SLMBP3	3.2554
JOINTLESS	TM5	TAGL1	5.8752
SLMBP24	TM5	TM4	1.2281
SLMBP24	TM5	SLMBP3	4.0883
SLMBP24	TM5	TAGL1	3.9253
RIN	TM5	RIN	1.0555
TM5	TM5	SLMBP3	6.4053
TM5	TM5	TAGL1	12.3512
RIN	TM5	SLMBP21	7.2945
RIN	TM5	TAG1	3.1023
RIN	TM5	JOINTLESS	8.4030
LeMADS1	TM5	JOINTLESS	23.8938
SLMBP21	TM5	SLMBP24	66.3126
SLMBP20	TM5	TM5	2.1237
SLMBP20	TM5	SLMBP18	1.1085
TM4	TM5	SLMBP18	1.2251
RIN	TM5	SLMBP7	2.7559
SLMBP18	TM5	JOINTLESS	17.4772
JOINTLESS	TM5	TM3	3.3672
TM3	TM5	SLMBP24	47.0260
TAGL11	TM5	RIN	1.2811
TAGL11	TM5	SLMBP24	25.9506
SLMBP21	TM5	TAG1	1.2399
JOINTLESS	TM5	TAG1	1.2986
TAGL1	TM5	RIN	3.6216
TAGL1	TM5	SLMBP21	4.4401
JOINTLESS	TM5	SLMBP21	29.2574
JOINTLESS	TM5	SLMBP24	2.2018
SLMBP24	TM5	TM5	27.9458

Ara-PIN yeast-three hybrid

BD	pTFT	AD	Miller units
AGL42	SEP3	AGL42	1.2075
SOC1	SEP3	SOC1	1.1860
AG	SEP3	AG	1.2991
AGL24	SEP3	AGL24	1.8335
SEP1	SEP3	AP1	4.5109
SEP1	SEP3	AGL42	1.1927
SEP1	SEP3	AG	2.7421
SEP1	SEP3	AGL24	1.7476
SHP1	SEP3	SEP2	1.8169
SEP3	SEP3	SEP2	1.4766
SEP4	SEP3	SEP3	1.2445
AGL42	SEP3	SEP3	1.5138
SEP3	SEP3	PI	1.1444
SEP4	SEP3	CAL	0.7348
SEP4	SEP3	SHP2	1.3513
SEP4	SEP3	AP3	1.3707
AG	SEP3	AP1	1.4622
AG	SEP3	CAL	1.2106
CAL	SEP3	SHP1	0.9335
CAL	SEP3	SHP2	1.2098
CAL	SEP3	AP3	1.2040
FUL	SEP3	AP3	1.0744
SOC1	SEP3	SEP4	1.3110
SOC1	SEP3	CAL	1.1959
SOC1	SEP3	AGL42	1.2344
SOC1	SEP3	AG	1.6490
SOC1	SEP3	SHP2	1.5425
SOC1	SEP3	AP3	1.3527
STK	SEP3	SEP2	1.2976
STK	SEP3	AG	1.2320
STK	SEP3	SHP2	3.0045
STK	SEP3	AP3	1.5780
STK	SEP3	SVP	1.6146
AG	SEP3	AP3	1.6690
SHP1	SEP3	SEP1	1.4986
SHP1	SEP3	PI	1.2792
SHP2	SEP3	SEP1	1.6271
SHP2	SEP3	SEP2	1.6554
SHP2	SEP3	SEP3	2.7446
SHP2	SEP3	AP3	1.0740
SHP2	SEP3	SVP	1.6277
PI	SEP3	AG	1.5042
AP3	SEP3	PI	1.2026
SVP	SEP3	SEP2	1.7551
SVP	SEP3	CAL	1.4917
SVP	SEP3	AG	1.6755
AGL24	SEP3	AGL42	1.6767
AGL24	SEP3	STK	1.3668
AGL24	SEP3	PI	1.2662
AP1	SEP3	AP1	1.0453
SHP1	SEP3	SHP1	1.0907
AP3	SEP3	AP3	1.9886
FUL	SEP3	SEP1	1.1132
SEP2	SEP3	SOC1	1.0187
SEP3	SEP3	AP1	2.4954
SEP3	SEP3	FUL	3.2264
SEP3	SEP3	STK	2.1864
AP3	SEP3	SEP3	2.6727
AP1	SEP3	CAL	1.2379
AP1	SEP3	FUL	2.9873
AP1	SEP3	AGL42	0.7764
SOC1	SEP3	AP1	1.3938
SVP	SEP3	AP1	4.1027
SOC1	SEP3	FUL	3.3447
FUL	SEP3	PI	1.1091
FUL	SEP3	AG	2.4469
AGL42	SEP3	SHP2	1.5940
AGL42	SEP3	AP3	1.1268
SHP1	SEP3	SOC1	1.0913
SVP	SEP3	AGL42	2.0690
SOC1	SEP3	SVP	3.0541
SVP	SEP3	FUL	2.2460
SOC1	SEP3	AGL24	1.9777
AP3	SEP3	AGL24	1.2139
AGL24	SEP3	SHP1	2.1492

ancE mediated yeast-three hybrid

BD	pTFT	AD	Miller
AP1	ancE	PI	1.5326
FLC	ancE	AGL24	0.6566
AG	ancE	AP3	1.6977
AG	ancE	PI	1.5577

post-PIN yeast-three hybrid

BD	pTFT	AD	Miller units
gSEP4	gSEP3	gSEP4	0.9060
geuAP1	gSEP3	geuAP1	1.7204
gFUL-LIKE	gSEP3	gFUL-LIKE	0.8206
gFBP22	gSEP3	gFBP22	1.9993
geuAG	gSEP3	geuAG	1.7234
gSVP	gSEP3	gSVP	2.9187
gAGL24	gSEP3	gAGL24	1.1968
gSEP1/2	gSEP3	gFBP9	1.4436
gSEP1/2	gSEP3	gSVP	1.3615
gSEP4	gSEP3	gFUL-LIKE	1.3580
gSEP4	gSEP3	gTM3	3.4796
gSEP4	gSEP3	gSTK	1.6262
gSEP4	gSEP3	gPI	2.2699
gSEP4	gSEP3	gTM6	2.4586
gFBP9	gSEP3	gFBP22	3.6328
geuAP1	gSEP3	gSEP1/2	2.0662
geuAP1	gSEP3	gFUL-LIKE	1.6243
geuAP1	gSEP3	gFUL	2.4867
geuAP1	gSEP3	gSTK	1.2617
geuAP1	gSEP3	gSHP	1.5401
geuAP1	gSEP3	gPI	2.6929
geuAP1	gSEP3	geuAP3	1.8759
geuAP1	gSEP3	gAGL24	1.1102
gFUL-LIKE	gSEP3	gSEP3	2.1377
gFUL-LIKE	gSEP3	geuAG	1.2758
gFUL-LIKE	gSEP3	gPI	1.9426
gFUL-LIKE	gSEP3	geuAP3	0.9902
gFUL-LIKE	gSEP3	gSVP	0.3144
gFUL	gSEP3	gTM3	2.1789
gFUL	gSEP3	gPI	0.4435
gFBP22	gSEP3	gSTK	1.6326
gFBP22	gSEP3	gSHP	1.5757
gFBP22	gSEP3	gPI	1.7142
gFBP22	gSEP3	geuAP3	2.0326
gFBP22	gSEP3	gAGL24	1.6674
gTM3	gSEP3	gSHP	0.7698
gTM3	gSEP3	geuAP3	0.9017
gSTK	gSEP3	gSEP1/2	2.5968
geuAG	gSEP3	gSEP4	2.0578
geuAG	gSEP3	gFBP9	1.6352
geuAG	gSEP3	geuAP1	1.8634
geuAG	gSEP3	gFUL	1.4611
geuAG	gSEP3	gTM3	1.2803
geuAG	gSEP3	gSTK	2.1377
geuAG	gSEP3	gPI	0.9358
geuAG	gSEP3	geuAP3	3.4953
geuAG	gSEP3	gTM6	0.7798
geuAP3	gSEP3	gFUL	2.9442
geuAP3	gSEP3	gSTK	1.3557
geuAP3	gSEP3	gPI	1.8142
geuAP3	gSEP3	gAGL24	1.2603
gSVP	gSEP3	gFUL	1.8505
gSVP	gSEP3	gFBP22	1.2843
gSVP	gSEP3	gSTK	0.9262
gSVP	gSEP3	geuAG	1.6898
gSVP	gSEP3	gSHP	1.5361
gSVP	gSEP3	gPI	1.4889
gAGL24	gSEP3	gSEP4	1.2337
gAGL24	gSEP3	gFUL-LIKE	2.1308
gAGL24	gSEP3	gFUL	1.8893
gAGL24	gSEP3	gSTK	1.3108
gAGL24	gSEP3	geuAG	1.4813
gAGL24	gSEP3	gSHP	2.3981
gAGL24	gSEP3	gPI	2.0139
gAGL24	gSEP3	gTM6	1.5365
gAGL24	gSEP3	gSVP	1.2391
gSEP3	gSEP3	gSEP3	4.9834
gSEP3	gSEP3	gFBP22	17.6449
gSEP3	gSEP3	gTM3	45.8710
gSEP3	gSEP3	geuAP3	6.8156
gSEP1/2	gSEP3	gSEP3	7.1308
gSEP4	gSEP3	gSEP3	3.7084
gSEP4	gSEP3	gFBP9	2.6947
gSEP4	gSEP3	gFUL	1.9939
gSEP4	gSEP3	geuAP3	3.0974
gSEP4	gSEP3	gSVP	2.2004
geuAP1	gSEP3	gSEP3	8.0558
geuAP1	gSEP3	gFBP22	14.8465
gFUL	gSEP3	gSEP3	7.9296
gFBP22	gSEP3	gFUL-LIKE	1.5150
gSTK	gSEP3	gSEP3	2.8326
geuAG	gSEP3	gSEP3	9.6188
geuAG	gSEP3	gSEP1/2	5.1958
geuAG	gSEP3	gFBP22	6.0135
gSEP1/2	gSEP3	gSHP	2.0857
gSEP4	gSEP3	gSHP	2.8788
gSVP	gSEP3	gSEP3	1.4890
gAGL24	gSEP3	gSEP3	2.0215
gFBP9	gSEP3	gSEP3	3.4565

post-PIN yeast-three hybrid

BD	pTFT	AD	Miller units
pSEP1/2/4	pSEP3	pSOC1	4.7141
pSEP1/2/4	pSEP3	pSTK	1.6764
pSEP1/2/4	pSEP3	pAG	1.9685
pSTK	pSEP3	pSOC1	2.1229
pAG	pSEP3	pPI	7.4232
pAG	pSEP3	pAP3	17.9637
pAP1	pSEP3	pSEP3	2.8688
pAP1	pSEP3	pSEP1/2/4	1.3677
pAP1	pSEP3	pSOC1	20.0725
pAP1	pSEP3	pSVP/AGL24	2.4172
pSOC1	pSEP3	pSEP3	5.7485

Supp.Table 2. Measurements of β-galactoside (ONPG) assays (miller units) of yeast-two hybrid assays.

ancE-AraPIN yeast-two hybrid		
AD	BD	Miller units
ancE	SHP1	1.4355
ancE	SEP3	4.2861
ancE	AG	13.0516
ancE	AGL42	1.4720
ancE	SVP	26.4150
ancE	SHP2	1.7318
ancE	SEP4	4.4172
ancE	AGL24	2.2399
ancE	SEP2	1.2930
ancE	STK	1.2495
ancE	AP1	9.0663
ancE	FUL	3.9957
ancE	CAL	0.7984
ancE	SEP1	8.5086
ancE	SOC1	7.3492
AP3	ancE	1.5854
PI	ancE	2.0318

ancE-prePIN yeast-two hybrid		
AD	BD	Miller units
ancE	pSEP3	17.6556
ancE	pSTK	7.6580
ancE	pAP3	1.3347
ancE	pAP1	3.4409
ancE	pSEP1/2/4	2.5664
ancE	pSOC	2.9496
ancE	pAG	19.2507
pSVP24	ancE	1.9269
pPI	ancE	1.4556

ancB-AraPIN yeast-two hybrid		
AD	BD	Miller units
FUL	ancB	4.4822
PI	ancB	0.7998
AGL24	ancB	2.0240
AG	ancB	0.6001
SCO1	ancB	0.6215

ancB-prePIN yeast-two hybrid		
AD	BD	Miller units
ancB	pAP1	0.8114
pPI	ancB	2.5521
pSEP3	ancB	16.3248

mutSEP3-AraPIN yeast-two hybrid		
AD	BD	Miller units
24	mutSEP3	0.3708
SVP	mutSEP3	199.8119
SOC1	mutSEP3	4.2290
AP1	mutSEP3	1.8544
AP3	mutSEP3	0.8087
SEP4	mutSEP3	3.6692
FUL	mutSEP3	3.1584
CAL	mutSEP3	0.9869
PI	mutSEP3	0.4362
AG	mutSEP3	2.7990
mutSEP3	AGL42	0.5224
mutSEP3	SHP2	0.3387
mutSEP3	SEP3	0.3109

pSEP3-AraPIN yeast-two hybrid		
AD	BD	Miller units
pSEP3	AG	20.3800
SVP	pSEP3	43.6690
pSEP3	SOC1	1.3840
pSEP3	SHP1	1.4792
pSEP3	SHP2	3.1662
pSEP3	SEP1	4.3493
pSEP3	SEP2	1.4661
pSEP3	FUL	2.2418
pSEP3	AP1	5.3363

pSEP3-prePIN yeast-two hybrid		
AD	BD	Miller units
pSEP3	pSEP3	3.2347
pSOC1	pSEP3	6.4646
pSEP3	pSEP1/2/4	7.5711
pSEP3	pSTK	18.1273
pSEP3	pAG	3.9811
pSEP3	pPI	1.7070

pSEP3-ePIN yeast-two hybrid		
AD	BD	Miller units
pSEP3	ancE	21.9319
pSEP3	ancB	16.3248
pSEP3	ancCD	13.6592

pAP3-AraPIN yeast-two hybrid		
AD	BD	Miller units
pAP3	AP1	0.7773
pAP3	AP3	1.2700
pAP3	AGL24	0.8202
pAP3	AGL42	0.9893
pAP3	SHP2	0.7864
SEP2	pAP3	0.7847
SEP4	pAP3	0.5962

pAP3-ePIN yeast-two hybrid		
AD	BD	Miller units
preAP3	ancCD	1.2222
preAP3	ancE	1.4691

pSVP24-prePIN yeast-two hybrid		
AD	BD	Mille units
pSVP24	pAG	0.7390

mutSVP-yeast-two hybrid		
AD	BD	Miller units
ins 80 M	SOC1	10.5676
del 89 V	SOC1	1.6669
Q127R	SOC1	15.9615
del 175-196	SOC1	1.8228
ins 220Y-221D	SOC1	8.1497
ins 80 M	SEP3	14.0166
del 89 V	SEP3	41.7546
Q127R	SEP3	10.7345
del 175-196	SEP3	2.3119
ins 220Y-221D	SEP3	30.4590
ins 80 M	FLC	1.3411
del 89 V	FLC	2.0730
Q127R	FLC	5.2016
del 175-196	FLC	0.4923
ins 220Y-221D	FLC	4.0204

SEP3-AraPIN yeast-two hybrid		
AD	BD	Miller units
SEP3	AGL24	2.7904
AP1	SEP3	0.7357
FUL	SEP3	0.9821
SOC1	SEP3	8.2547
STK	SEP3	1.0345
AG	SEP3	32.3479
AP3	SEP3	0.8612
SVP	SEP3	44.9211

AP3-AraPIN yeast-two hybrid		
AD	BD	Miller units
AP3	AP3	1.3945
AP3	SEP3	0.8612
AGL42	AP3	10.9056
AP3	SVP	4.0007
AP3	AGL24	5.2608

AP3-prePIN yeast-two hybrid		
AD	BD	Miller units
preSVP24	AP3	0.8523

AP3-ePIN yeast-two hybrid		
AD	BD	Miller units
preAP3	ancCD	1.2222
preAP3	ancE	1.4691

SVP-AraPIN yeast-two hybrid		
AD	BD	Miller units
SVP	SEP1	73.7755
SVP	SEP3	44.9211
SVP	AP1	31.0896
FUL	SVP	1.3435
AGL42	SVP	0.8367
SOC1	SVP	20.4072
PI	SVP	3.3190
AP3	SVP	4.0007

SVP-prePIN yeast-two hybrid		
AD	BD	Miller units
SVP	pSEP3	194.5758
SVP	pSEP124	40.2307
SVP	pSTK	6.8697
SVP	pAG	74.1934
pSOC	SVP	4.6027

ePIN-yeast-two hybrid		
AD	BD	Miller units
ancB	ancB	11.4274
ancE	ancE	1.6937
ancB	ancC/D	117.5542
ancB	ancE	14.4305
ancE	ancC/D	5.5684

1 Unit is defined as the amount which hydrolyzes 1 micromol of ONPG to o-nitrophenol and D-galactose per min per cell (Miller 1972, Miller 1992).
